# Author Correction: Exome sequencing identifies breast cancer susceptibility genes and defines the contribution of coding variants to breast cancer risk

**DOI:** 10.1038/s41588-023-01549-x

**Published:** 2023-09-26

**Authors:** Naomi Wilcox, Martine Dumont, Anna González-Neira, Sara Carvalho, Charles Joly Beauparlant, Marco Crotti, Craig Luccarini, Penny Soucy, Stéphane Dubois, Rocio Nuñez-Torres, Guillermo Pita, Eugene J. Gardner, Joe Dennis, M. Rosario Alonso, Nuria Álvarez, Caroline Baynes, Annie Claude Collin-Deschesnes, Sylvie Desjardins, Heiko Becher, Sabine Behrens, Manjeet K. Bolla, Jose E. Castelao, Jenny Chang-Claude, Sten Cornelissen, Thilo Dörk, Christoph Engel, Manuela Gago-Dominguez, Pascal Guénel, Andreas Hadjisavvas, Eric Hahnen, Mikael Hartman, Belén Herráez, SGBCC Investigators, Benita Kiat-Tee Tan, Veronique Kiak Mien Tan, Su-Ming Tan, Geok Hoon Lim, Ern Yu Tan, Peh Joo Ho, Alexis Jiaying Khng, Audrey Jung, Renske Keeman, Marion Kiechle, Jingmei Li, Maria A. Loizidou, Michael Lush, Kyriaki Michailidou, Mihalis I. Panayiotidis, Xueling Sim, Soo Hwang Teo, Jonathan P. Tyrer, Lizet E. van der Kolk, Cecilia Wahlström, Qin Wang, John R. B. Perry, Javier Benitez, Marjanka K. Schmidt, Rita K. Schmutzler, Paul D. P. Pharoah, Arnaud Droit, Alison M. Dunning, Anders Kvist, Peter Devilee, Douglas F. Easton, Jacques Simard

**Affiliations:** 1 Centre for Cancer Genetic Epidemiology, Department of Public Health and Primary Care, University of Cambridge, Cambridge, UK; 2 Genomics Center, Centre Hospitalier Universitaire de Québec—Université Laval Research Center, Québec City, Quebec Canada; 3 Human Genotyping Unit-CeGen, Human Cancer Genetics Programme, Spanish National Cancer Research Centre (CNIO), Madrid, Spain; 4 Centre for Cancer Genetic Epidemiology, Department of Oncology, University of Cambridge, Cambridge, UK; 5 MRC Epidemiology Unit, Wellcome-MRC Institute of Metabolic Science, University of Cambridge, Cambridge, UK; 6 Institute of Medical Biometry and Epidemiology, University Medical Center Hamburg-Eppendorf, Hamburg, Germany; 7 Division of Cancer Epidemiology, German Cancer Research Center (DKFZ), Heidelberg, Germany; 8 Oncology and Genetics Unit, Instituto de Investigación Sanitaria Galicia Sur (IISGS), Xerencia de Xestion Integrada de Vigo-SERGAS, Vigo, Spain; 9 Cancer Epidemiology Group, University Cancer Center Hamburg (UCCH), University Medical Center Hamburg-Eppendorf, Hamburg, Germany; 10 Division of Molecular Pathology, The Netherlands Cancer Institute, Amsterdam, the Netherlands; 11 Gynaecology Research Unit, Hannover Medical School, Hannover, Germany; 12 Institute for Medical Informatics, Statistics and Epidemiology, University of Leipzig, Leipzig, Germany; 13 LIFE—Leipzig Research Centre for Civilization Diseases, University of Leipzig, Leipzig, Germany; 14 Cancer Genetics and Epidemiology Group, Instituto de Investigación Sanitaria de Santiago de Compostela (IDIS) Foundation, Complejo Hospitalario Universitario de Santiago, SERGAS, Santiago de Compostela, Spain; 15 Team ‘Exposome and Heredity,’ CESP, Gustave Roussy, INSERM, University Paris-Saclay, UVSQ, Villejuif, France; 16 Department of Cancer Genetics, Therapeutics and Ultrastructural Pathology, The Cyprus Institute of Neurology & Genetics, Nicosia, Cyprus; 17 Center for Familial Breast and Ovarian Cancer, Faculty of Medicine and University Hospital Cologne, University of Cologne, Cologne, Germany; 18 Center for Integrated Oncology (CIO), Faculty of Medicine and University Hospital Cologne, University of Cologne, Cologne, Germany; 19 Saw Swee Hock School of Public Health, National University of Singapore and National University Health System, Singapore City, Singapore; 20 Department of Surgery, National University Health System, Singapore City, Singapore; 21 Department of Pathology, Yong Loo Lin School of Medicine, National University of Singapore, Singapore City, Singapore; 22 Division of Gynaecology and Obstetrics, Klinikum rechts der Isar der Technischen Universität München, Munich, Germany; 23 Genome Institute of Singapore, Agency for Science, Technology and Research, Singapore City, Singapore; 24 Biostatistics Unit, The Cyprus Institute of Neurology & Genetics, Nicosia, Cyprus; 25 Breast Cancer Research Programme, Cancer Research Malaysia, Subang Jaya, Malaysia; 26 Department of Surgery, Faculty of Medicine, University of Malaya, UM Cancer Research Institute, Kuala Lumpur, Malaysia; 27 Family Cancer Clinic, The Netherlands Cancer Institute—Antoni van Leeuwenhoek hospital, Amsterdam, the Netherlands; 28 Division of Oncology, Department of Clinical Sciences Lund, Lund University, Lund, Sweden; 29 Metabolic Research Laboratory, Wellcome-MRC Institute of Metabolic Science, University of Cambridge, Cambridge, UK; 30 Human Genetics Group, Spanish National Cancer Research Centre (CNIO), Madrid, Spain; 31 Centre for Biomedical Network Research on Rare Diseases (CIBERER), Instituto de Salud Carlos III, Madrid, Spain; 32 Division of Psychosocial Research and Epidemiology, The Netherlands Cancer Institute—Antoni van Leeuwenhoek hospital, Amsterdam, the Netherlands; 33 Center for Molecular Medicine Cologne (CMMC), Faculty of Medicine and University Hospital Cologne, University of Cologne, Cologne, Germany; 34 Département de Médecine Moléculaire, Faculté de Médecine, Centre Hospitalier Universitaire de Québec Research Center, Laval University, Québec City, Quebec Canada; 35 Department of Pathology, Leiden University Medical Center, Leiden, the Netherlands; 36 Department of Human Genetics, Leiden University Medical Center, Leiden, the Netherlands; 37 Department of Breast Surgery, Singapore General Hospital, Singapore City, Singapore; 38 Division of Surgical Oncology, National Cancer Centre, Singapore City, Singapore; 39 Department of General Surgery, Sengkang General Hospital, Singapore City, Singapore; 40 Division of Breast Surgery, Department of General Surgery, Changi General Hospital, Singapore City, Singapore; 41 KK Breast Department, KK Women’s and Children’s Hospital, Singapore City, Singapore; 42 Department of General Surgery, Tan Tock Seng Hospital, Singapore City, Singapore; 43 Lee Kong Chian School of Medicine, Nanyang Technological University, Singapore City, Singapore; 44 Institute of Molecular and Cell Biology, Singapore City, Singapore

**Keywords:** Breast cancer, Genetics research, Epidemiology

Correction to: *Nature Genetics* 10.1038/s41588-023-01466-z, published online 17 August 2023.

In the version of the article initially published, in the sentence in the Abstract now reading “Associations were also observed for *LZTR1*, *ATRIP* and *BARD1* with *P* < 1 × 10^−4^”, “*ATRIP*” appeared incorrectly as “*ATR*”. This has now been corrected in the PDF and HTML versions of the article.

