# Supplementary material for: Exome sequencing identifies breast cancer susceptibility genes and defines the contribution of coding variants to breast cancer risk
Source: Nat Genet. 2023 Aug 17;55(9):1435–9. doi: 10.1038/s41588-023-01466-z (PMC10484782; doi:10.1038/s41588-023-01466-z)
Supplement: Supplementary file 1 — Supplementary Note, Supplementary Tables 1,2,4,10,14–17, 19 and Supplementary Methods. [file 41588_2023_1466_MOESM1_ESM.pdf]

nature genetics

Letter

https://doi.org/10.1038/s41588-023-01466-z

Exome sequencing identifies breast cancer susceptibility genes and defines the contribution of coding variants to breast cancer risk

In the format provided by the authors and unedited

Supplementary Material

Exome sequencing identifies breast cancer susceptibility genes and defines the contribution of coding variants to breast cancer risk

Naomi Wilcox, Martine Dumont, Anna González-Neira, Sara Carvalho, Charles Joly Beauparlant, Marco Crotti, Craig Luccarini, Penny Soucy, Stéphane Dubois, Rocio Nuñez-Torres, Guillermo Pita, Eugene J. Gardner, Joe Dennis, M. Rosario Alonso, Nuria Álvarez, Caroline Baynes, Annie Claude Collin-Deschesnes, Sylvie Desjardins, Heiko Becher, Sabine Behrens, Manjeet K. Bolla, Jose E. Castelao, Jenny Chang-Claude, Sten Cornelissen, Thilo Dörk, Christoph Engel, Manuela Gago-Dominguez, Pascal Guénel, Andreas Hadjisavvas, Eric Hahnen, Mikael Hartman, Belén Herráez, SGBCC Investigators, Audrey Jung, Renske Keeman, Marion Kiechle, Jingmei Li, Maria A. Loizidou, Michael Lush, Kyriaki Michailidou, Mihalis I. Panayiotidis, Xueling Sim, Soo Hwang Teo, Jonathan P. Tyrer, Lizet E. van der Kolk, Cecilia Wahlström, Qin Wang, John D. Perry, Javier Benitez, Marjanka K. Schmidt, Rita K. Schmutzler, Paul D.P. Pharoah, Arnaud Droit, Alison M. Dunning, Anders Kvist, Peter Devilee, Douglas F. Easton, Jacques Simard

Contents

Supplementary Note *1*

BCAC Study specific funding 1

BCAC Study specific acknowledgments 1

Supplementary Tables 1,2,4,10,14-17,19 .... *3*

Supplementary Table Legends 3,5-9, 11-13, 18 13

Supplementary Methods *14*

Variant calling in the Breast Cancer Association Consortium datasets 14

Contribution of PTVs to the Familial Relative Risk 16

Supplementary Note

BCAC Study specific funding

The **ABCS** study was supported by the Dutch Cancer Society [grants NKI 2007-3839; 2009 4363]. The BREast Oncology GAlician Network (**BREOGAN**) is funded by Acción Estratégica de Salud del Instituto de Salud Carlos III FIS PI12/02125/Cofinanciado and FEDER PI17/00918/Cofinanciado FEDER; Acción Estratégica de Salud del Instituto de Salud Carlos III FIS Intrasalud (PI13/01136); Programa Grupos Emergentes, Cancer Genetics Unit, Instituto de Investigacion Biomedica Galicia Sur. Xerencia de Xestion Integrada de Vigo-SERGAS, Instituto de Salud Carlos III, Spain; Grant 10CSA012E, Consellería de Industria Programa Sectorial de Investigación Aplicada, PEME I + D e I + D Suma del Plan Gallego de Investigación, Desarrollo e Innovación Tecnológica de la Consellería de Industria de la Xunta de Galicia, Spain; Grant EC11-192. Fomento de la Investigación Clínica Independiente, Ministerio de Sanidad, Servicios Sociales e Igualdad, Spain; and Grant FEDER-Innterconecta. Ministerio de Economia y Competitividad, Xunta de Galicia, Spain. The **CECILE** study was supported by Fondation de France, Institut National du Cancer (INCa), Ligue Nationale contre le Cancer, Agence Nationale de Sécurité Sanitaire, de l'Alimentation, de l'Environnement et du Travail (ANSES), Agence Nationale de la Recherche (ANR). The **GC-HBOC** (German Consortium of Hereditary Breast and Ovarian Cancer) is supported by the German Cancer Aid (grant no 110837 and 70114178, coordinator: Rita K. Schmutzler, Cologne) and the Federal Ministry of Education and Research, Germany (grant no 01GY1901). This work was also funded by the European Regional Development Fund and Free State of Saxony, Germany (LIFE - Leipzig Research Centre for Civilization Diseases, project numbers 713-241202, 713-241202, 14505/2470, 14575/2470). The **GESBC** was supported by the Deutsche Krebshilfe e. V. [70492] and the German Cancer Research Center (DKFZ). The **HABCS** study was supported by the Claudia von Schilling Foundation for Breast Cancer Research, by the Lower Saxonian Cancer Society, by the Rudolf Bartling Foundation and by the German Research Foundation. The **MARIE** study was supported by the Deutsche Krebshilfe e.V. [70-2892-BR I, 106332, 108253, 108419, 110826, 110828], the Hamburg Cancer Society, the German Cancer Research Center (DKFZ) and the Federal Ministry of Education and Research (BMBF) Germany [01KH0402]. The **MASTOS** study was supported by "Cyprus Research Promotion Foundation" grants 0104/13 and 0104/17, and the Cyprus Institute of Neurology and Genetics. **MYBRCA** is funded by research grants from the Wellcome Trust (v203477/Z/16/Z), the Malaysian Ministry of Higher Education (UM.C/HlR/MOHE/06) and Cancer Research Malaysia. **SEARCH** is funded by Cancer Research UK [C490/A10124, C490/A16561] and supported by the UK National Institute for Health Research Biomedical Research Centre at the University of Cambridge. The University of Cambridge has received salary support for PDPP from the NHS in the East of England through the Clinical Academic Reserve. **SGBCC** is funded by the National Research Foundation Singapore, NUS start-up Grant, National University Cancer Institute Singapore (NCIS) Centre Grant, Breast Cancer Prevention Programme, Asian Breast Cancer Research Fund and the NMRC Clinician Scientist Award (SI Category). Population-based controls were from the Multi-Ethnic Cohort (MEC) funded by grants from the Ministry of Health, Singapore, National University of Singapore and National University Health System, Singapore.

BCAC Study specific acknowledgments

ABCS thanks the Blood bank Sanquin, The Netherlands. The **BREOGAN** study would not have been possible without the contributions of the following: Manuela Gago-Dominguez, Jose Esteban Castelao, Angel Carracedo, Victor Muñoz Garzón, Alejandro Novo Domínguez, Maria Elena Martinez, Sara Miranda Ponte, Carmen Redondo Marey, Maite Peña Fernández, Manuel Enguix Castelo, Maria Torres, Manuel Calaza (BREOGAN), José Antúnez, Máximo Fraga and the staff of the Department of Pathology and Biobank of the University Hospital Complex of Santiago-CHUS, Instituto de

Investigación Sanitaria de Santiago, IDIS, Xerencia de Xestion Integrada de Santiago-SERGAS; Joaquín González-Carreró and the staff of the Department of Pathology and Biobank of University Hospital Complex of Vigo, Instituto de Investigacion Biomedica Galicia Sur, SERGAS, Vigo, Spain. **HABCS** thanks Michael Bremer, Johann H. Karstens, Peter Schürmann, Natalia Bogdanova, Hans Christiansen, Tjoung-Won Park-Simon and Peter Hillemanns. **MARIE** thanks Petra Seibold, Nadia Obi, Sabine Behrens, Ursula Eilber and Muhabbet Celik. **MASTOS** thanks all the study participants and express appreciation to the doctors: Yiola Marcou, Eleni Kakouri, Panayiotis Papadopoulos, Simon Malas and Maria Daniel, as well as to all the nurses and volunteers who provided valuable help towards the recruitment of the study participants. **MYBRCA** thanks study participants and research staff (particularly Patsy Ng, Nurhidayu Hassan, Yoon Sook-Yee, Daphne Lee, Lee Sheau Yee, Phuah Sze Yee and Norhashimah Hassan) for their contributions and commitment to this study. We thank the **SEARCH** and EPIC teams. **SGBCC** thanks the participants and all research coordinators for their excellent help with recruitment, data and sample collection.

Supplementary Tables 1,2,4,10,14-17,19

Supplementary Table 1: **Breast Cancer Association Consortium (BCAC) studies included in the analysis.**

Dataset	Study	Country	Total	Controls	Cases
BRIDGES	ABCS	Netherlands	1421	716	705
BRIDGES	BREOGAN	Spain	549	305	244
BRIDGES	CECILE	France	744	371	373
BRIDGES	GESBC	Germany	347	138	209
BRIDGES	HABCS	Germany	742	456	286
BRIDGES	MARIE	Germany	693	398	295
BRIDGES	MYBRCA	Malaysia	921	455	466
BRIDGES	SGBCC	Singapore	1244	622	622
PERSPECTIVE	GC-HBOC	Germany	2590	1379	1211
PERSPECTIVE	MASTOS	Cyprus	736	397	339
PERSPECTIVE	SEARCH	UK	6661	3001	3660
			**16648**	**8238**	**8410**

Supplementary Table 2: **Summary of numbers of cases and controls used in the analysis, as well as median age of participants.** ER=oestrogen receptor; there was no ER data available for the UK biobank. Family history was a selection criterion for case inclusion in BRIDGES and PERSPECTIVE. The variable median age for cases is the median diagnosed age and for controls is the median age at the first assessment centre visit (UK Biobank) or observation (BRIDGES and PERSPCTIVE).

	**Female Cases**	**Female Controls**	**Male Cases**	**Male Controls**
**Total**	**26368**	**217673**	**94**	**191820**
**BRIDGES**	**3200**	**3461**		
ER positive	2400			
ER negative	798			
ER status not available	2			
No family history	1982	1848		
family history	949	202		
family history not available	269	1411		
median age	*46*	*51*		
**PERSPECTIVE**	**5210**	**4777**		
ER positive	3924			
ER negative	982			
ER status not available	304			
No family history	2065	885		
family history	2149	227		
family history not available	996	3615		
median age	*49*	*57*		
**UK Biobank**	**17958**	**209435**	**94**	**191820**
No family history	14721	186602	79	171913
family history	3237	22833	15	19907
median age	*57*	*58*	*63*	*58*

Supplementary Table 4: **Association results for previously established breast cancer susceptibility genes**. Z-scores are from testing H0:β=ln(Odds Ratio)=0 (2-tailed).P-values are unadjusted for multiple testing. The counts are for female cases and controls only but odds-ratios and results are from incorporating males and family history data - see online methods.

		**Dataset Level Results**							**Meta-Analysis results**	
		**Controls***		**Cases***						
		**Non-Carriers**	**Carriers**	**Non-Carriers**	**Carriers**	**Odds Ratio**	**Z-score**	**P-value**	**Z-score**	**P-value**
CDH1	BCAC	8237	1	8407	3	1.98 (0.372, 10.5)	0.800	0.424	3.36	0.000776
	UKB	209427	8	17954	4	5.14 (1.97, 13.4)	3.34	0.000800		
PTEN	BCAC	8238	0	8404	6	7.03 (0.988, 50.1)	1.95	0.0515	0.82	0.412
	UKB	209434	1	17958	0	4.82e-13 (0, Inf)	-0.01	0.989		
RAD51C	BCAC	8234	4	8403	7	1.58 (0.591, 4.22)	0.91	0.362	0.67	0.504
	UKB	209370	65	17954	4	1.11 (0.58, 2.11)	0.31	0.759		
RAD51D	BCAC	8234	4	8401	9	2.06 (0.777, 5.46)	1.45	0.146	3.08	0.00210
	UKB	209353	82	17944	14	1.91 (1.2, 3.05)	2.72	0.00660		
STK11	BCAC	3461	0	3199	1	1.76e+15 (0, Inf)	0.01	0.993	-0.01	0.993
	UKB	209433	2	17958	0	4.63e-13 (0, Inf)	-0.01	0.989		
TP53	BCAC	3461	0	3198	2	3.58 (0.23, 55.8)	0.910	0.362	1.98	0.0481
	UKB	209435	0	17957	1	14.5 (0.733, 288)	1.76	0.079		

Supplementary Table 10: **Combined analysis of MAP3K1 PTVs and common variants at the MAP3K1 locus.** P-values are unadjusted for multiple testing and from a 2-tailed test of log(OR)=0.

Model	Regression	SNP	SNP OR (95%CI), p-value	$MAP3K1_{burden}$ OR (95% CI), p-value		
**M0**	Case ~ $MAP3K1_{burden}$			4.98 (2.28, 10.89), $p=5.6x10^{-5}$		
					**LRT p-value M2 vs M1**	**LRT p-value M2 vs M0**
**$M1_i$**	Case ~ SNP_i	rs62355902	1.17 (1.14, 1.21), $p=1.2x10^{-28}$			
		rs984113	0.95 (0.93, 0.97), $p=6.5x10^{-6}$			
		rs112497245	1.18 (1.13, 1.23), $p=4.2x10^{-15}$			
**$M2_i$**	Case ~ SNP_i + $MAP3K1_{burden}$	rs62355902	1.17 (1.14, 1.21), $p=1.2x10^{-28}$	4.96 (2.27, 10.83), $p=6.0x10^{-5}$	0.000435	$6.89x10^{-28}$
		rs984113	0.95 (0.93, 0.97), $p=6.5x10^{-6}$	4.99 (2.28, 10.89), $p=5.6x10^{-5}$	0.000417	$6.78x10^{-6}$
		rs112497245	1.18 (1.13, 1.22), $p=4.4x10^{-15}$	4.97 (2.27, 10.85), $p=5.8x10^{-5}$	0.000429	$1.37x10^{-14}$
					**LRT p-value M4 vs M3**	**LRT p-value M4 vs M0**
**M3**	Case ~ SNP_1 + SNP_2 + SNP_3	rs62355902	1.16 (1.12, 1.20), $p=1.9x10^{-18}$			
		rs984113	0.93 (0.91, 0.95), $p=2.15x10^{-9}$			
		rs112497245	1.07 (1.02, 1.12), $p=5.0x10^{-3}$			
**M4**	Case ~ SNP_1 + SNP_2 + SNP_3 + $MAP3K1_{burden}$	rs62355902	1.16 (1.12, 1.20), $p=2.0x10^{-18}$	4.95 (2.27, 10.82), $p=6.05x10^{-5}$	0.000439	$6.81x10^{-35}$
		rs984113	0.93 (0.91, 0.95), $p=2.16x10^{-9}$			
		rs112497245	1.07 (1.02, 1.12), $p=5.0x10^{-3}$			

Supplementary Table 14: **GSEA results for KEGG, REACTOME and BIOCARTA pathways**, **using gene rankings based on Z scores for the gene PTV burden meta-analysis.** Excluding BRCA1, BRCA2, CHEK2, ATM and PALB2. All results with q<0.1 are listed.

Pathway	Set Size	Enrichment Score	NES=	p-value	q-value	rank	core_enrichment
BIOCARTA_NFKB_PATHWAY	21	0.750	2.09	1.31E-05	0.0264	1045	MAP3K1/TRAF6/MYD88/MAP3K7/MAP3K14
REACTOME_DNA_DOUBLE_STRAND_BREAK_RESPONSE	35	0.611	1.92	0.000101	0.0736	1185	BARD1/BAP1/HERC2/BABAM1/TP53/UIMC1/EYA1/SMARCA5/PPP5C
BIOCARTA_CD40_PATHWAY	15	0.778	1.99	0.000125	0.0736	1061	MAP3K1/TRAF6/MAP3K14/CD40
REACTOME_PEPTIDE_HORMONE_BIOSYNTHESIS	11	0.817	1.91	0.000146	0.0736	880	INHBE/PCSK1/LHB

Supplementary Table 15: **Estimates by gene of the posterior probability of being disease associated, effect sizes and proportion of the familial relative risk (FRR) explained.** This is based on the best fitting model (see online methods). Genes listed have %FRR>0.01. a – estimated allele frequency, b-estimated FRR due to PTVs, c – estimated percentage of the FRR due to PTVs.

	**log-likelihood**	**posterior probability**	**Posterior mean β**	**Posterior mean e^{β}**	**p[a]**	**λ[b]**	**%FRR[c]**
BRCA1	192.91	1	2.10	8.19	0.0006394	1.0376	5.3280
BRCA2	360.07	1	1.69	5.45	0.00102	1.0202	2.8780
PALB2	99.95	1	1.32	3.76	0.00064	1.0050	0.7220
CHEK2	94.5	1	0.85	2.35	0.00238505	1.0048	0.6910
ATM	42.49	1	0.80	2.23	0.00117034	1.0019	0.2810
MAP3K1	12.9	0.999	1.49	4.59	6.10E-05	1.0009	0.1360
CUL9	3.81	0.178	0.57	1.80	0.00279903	1.0004	0.0560
ATRIP	5.64	0.573	0.79	2.25	0.00015672	1.0002	0.0250
BAP1	5.13	0.446	1.21	3.55	3.90E-05	1.0001	0.0200
BARD1	4.33	0.266	0.61	1.87	0.00021428	1.0001	0.0150
LZTR1	5	0.413	0.36	1.44	0.00098919	1.0001	0.0130
USP6	1.71	0.026	0.57	1.86	0.00268396	1.0001	0.0130
MARCHF10	2.49	0.055	0.56	1.81	0.00180491	1.0001	0.0130
PCDHGB3	4.84	0.375	0.35	1.43	0.00110008	1.0001	0.0120
SEC62	3.14	0.1	1.65	6.49	8.56E-06	1.0001	0.0120
OSTN	2.59	0.06	0.89	2.62	4.27E-05	1.0001	0.0100
**All genes**						**1.076**	**10.61**
**All genes except *BRCA1*, *BRCA2*, *ATM, CHEK2* and *ATM***						**1.0068**	**0.974**

.

Supplementary Table 16: **Heritability analysis for subsets of genes -** estimating the contribution of genes in each list to the FRR of breast cancer.

	N genes	Top 5 genes (*ATM, BRCA1, BRCA2, CHEK2, PALB2*) included	alpha	eta	All genes			Excluding top 5 known genes		
					Sum(log-lik)	λ	%FRR	Sum(log-lik)	λ	%FRR
**Breast cancer driver genes (whole list, Fachal et al, 2020)**	278	*BRCA1, BRCA2, CHEK2, ATM, PALB2*	0.188	1.59	795	1.074	10.2	11.5	1.003	0.498
**High confidence breast cancer target genes (Level 1 target genes, ST6, Fachal et al, 2020)**	191	*BRCA2, CHEK2, ATM*	0.154	1.93	501	1.029	4.13	8.73	1.002	0.303
**Breast cancer driver genes with INQUISIT score 1 (Fachal et al, 2020)**	35	*BRCA1, BRCA2, CHEK2, ATM*	0.532	1.28	700	1.067	9.38	10.0	1.002	0.272
**Rahman Cancer Predisposition Genes (CPGs)**	114	*BRCA1, BRCA2, CHEK2, ATM, PALB2*	0.218	1.45	787	1.072	9.99	1.71	1.001	0.216
**COSMIC TSGs**	320	*BRCA1, BRCA2, CHEK2, ATM, PALB2*	0.196	1.92	799	1.074	10.3	16.2	1.004	0.639
**REACTOME_DNA_DOUBLE_STRAND_BREAK_REPAIR**	125	*BRCA1, BRCA2, CHEK2, ATM, PALB2*	0.253	1.73	795	1.072	10.1	10.5	1.003	0.366
**REACTOME_HOMOLOGOUS_DNA_PAIRING_AND_STRAND_EXCHANGE**	43	*BRCA1, BRCA2, CHEK2, ATM, PALB2*	0.389	1.59	700	1.067	9.32	6.87	1.001	0.210
**REACTOME_HDR_THROUGH_HOMOLOGOUS_RECOMBINATION_HRR**	60	*BRCA1, BRCA2, ATM, PALB2*	0.279	1.55	698	1.067	9.31	5.09	1.001	0.203
**REACTOME_HOMOLOGY_DIRECTED_REPAIR**	97	*BRCA1, BRCA2, ATM, PALB2*	0.211	1.61	698	1.067	9.36	6.38	1.002	0.259
**REACTOME_G2_M_DNA_DAMAGE_CHECKPOINT**	62	*BRCA1, BRCA2, ATM*	0.316	1.88	336	1.046	6.52	9.27	1.002	0.239
**BIOCARTA_NFKB_PATHWAY**	29		0.462	1.68	13.1	1.004	0.556	13.1	1.004	0.556
**REACTOME_DNA_DOUBLE_STRAND_BREAK_RESPONSE**	45	*BRCA1, CHEK2, ATM*	0.400	1.52	337	1.046	6.55	9.50	1.002	0.232
**BIOCARTA_CD40_PATHWAY**	16		0.628	1.43	14.2	1.005	0.657	14.2	1.005	0.657
**REACTOME_PEPTIDE_HORMONE_BIOSYNTHESIS**	11		1	5.87	0.606	1.00006	0.00922	0.606	1.00006	0.00922

Supplementary Table 17. **Ethics committees for the studies included.**

Study	Acronym	Country	Approval Committee(s)
Amsterdam Breast Cancer Study	ABCS	Netherlands	Leiden University Medical Center (LUMC) Commissie Medische Ethiek; Protocol Toetsingscommissie van Het Nederlands Kanker Instituut-Antoni van Leeuwenhoek Ziekenhuis
Breast Oncology Galicia Network	BREOGAN	Spain	Comité Autonómico de Ética de la Investigación de Galicia
CECILE Breast Cancer Study	CECILE	France	Comité Consultatif de Protection des Personnes dans la Recherche Biomédicale de Bicêtre (Le Kremlin-Bicêtre FR-94270)
Genetic Epidemiology Study of Breast Cancer by Age 50	GESBC	Germany	Medizinische Fakultat Heidelberg Ethikkommission
Hannover Breast Cancer Study	HABCS	Germany	Medizinische Hochschule Hannover Ethik-Kommission
Mammary Carcinoma Risk Factor Investigation	MARIE	Germany	Medizinische Fakultat Heidelberg Ethikkommission; Ethik-Kommission der Arztekammer Hamburg
Malaysian Breast Cancer Genetic Study	MYBRCA	Malaysia	University Malaya Medical Centre Medical Ethics Committee; Ramsay Sime Darby Independent Ethics Committee
Singapore Breast Cancer Cohort	SGBCC	Singapore	Cases: National Health Group (NHG) Domain Specific Review Board (DSRB); SingHealth Centralised Institutional Review Board (CIRB). Controls: National University of Singapore (NUS) IRB.
German Consortium for Hereditary Breast & Ovarian Cancer	GC-HBOC	Germany	Ethik-Kommission der Medizinischen Fakultat der Universitat zu Koln
Cyprus Breast Cancer Case Control Study	MASTOS	Cyprus	Cyprus National Bioethics committee
Study of Epidemiology and Risk factors in Cancer Heredity	SEARCH	UK	Multi Centre Research Ethics Committee (MREC)
UK Biobank		UK	North West Multi-centre Research Ethics Committee (MREC)

Supplementary Table 19.: **Comparison of the primary meta-analysis method with that of Han and Eskin[1].** All p-values are unadjusted for multiple testing. $\hat{\mu}$ - mean effect estimate, $\hat{\tau}$ - between study variance, S_HET - test statistic with $\hat{\tau}$ =0, i.e., the test statistic testing for heterogeneity, S_FE - contribution of the mean effect, S - test statistic = S_FE + S_HET.

Gene	Han and Eskin											*Our method*	
	$\hat{\mu}$	$\hat{\tau}$	**S_HET**	**S_FE**	**S**	**p-value**	**B**	**SE**	**Odds Ratio**	**OR LB**	**OR UB**	***Z-score***	***p-value***
ATM	0.858	0	0	107	107	3.51E-24	0.858	0.0830	2.36	2.00	2.77	*10.3*	*4.44E-25*
ATRIP	0.957	0	0	17.0	17.0	0.000120	0.957	0.232	2.60	1.65	4.10	*4.07*	*4.70E-05*
BARD1	0.768	0	0	15.4	15.4	0.000269	0.768	0.196	2.16	1.47	3.16	*3.96*	*7.56E-05*
BRCA1	2.31	0.00894	0.00362	443	443	2.81E-97	2.31	0.110	10.1	8.15	12.5	*20.9*	*4.14E-97*
BRCA2	1.85	0.00364	0.00266	891	891	1.61E-194	1.85	0.0621	6.37	5.64	7.19	*29.2*	*6.82E-188*
CHEK2	0.927	0	0	279	279	1.45E-61	0.927	0.0555	2.53	2.27	2.82	*16.7*	*1.13E-62*
LZTR1	0.438	0	0	16.2	16.2	0.000180	0.438	0.109	1.55	1.25	1.92	*4.11*	*4.02E-05*
MAP3K1	1.76	0	0	36.9	36.9	5.49E-09	1.76	0.289	5.80	3.29	10.2	*6.08*	*1.21E-09*
PALB2	1.43	0	0	254	254	4.05E-56	1.43	0.0900	4.19	3.52	5.00	*14.8*	*1.09E-49*

Supplementary Table Legends 3,5-9, 11-13, 18

See Excel spreadsheet for tables.

Supplementary Table 3**: Association results for PTVs and overall breast cancer**. All genes associated at P<0.001 in the meta-analysis are listed. Z-scores are from testing H0:β=ln(Odds Ratio)=0 (2-tailed). P-values are unadjusted for multiple testing. The counts are for female cases and controls only but odds-ratios and results are from incorporating males and family history data - see online methods.

Supplementary Table 5: **Association results for PTVs and overall breast cancer, restricting cases to age <=50.** All genes associated at P<0.001 in the meta-analysis are listed, followed by other genes that had P<0.0001 in ST3 for the analysis with all cases. Z-scores are from testing H0:β=ln(Odds Ratio)=0 (2-tailed). P-values are unadjusted for multiple testing. The counts are for female cases and controls only but odds-ratios and results are from incorporating males and family history data - see online methods.

Supplementary Table 6: **Association results for PTVs for breast cancer subtypes, based on the BCAC dataset**. Genes associated at P<0.001 for each analysis are included. Z-scores are from testing H0:β=ln(Odds Ratio)=0 (2-tailed). P-values are unadjusted for multiple testing. The counts are for female cases and controls only but odds-ratios and results are from incorporating males and family history data - see online methods.

Supplementary Table 7**: Association results for rare missense variants and overall breast cancer.** All genes associated at P<0.001 in the meta-analysis are listed. Z-scores are from testing H0:β=ln(Odds Ratio)=0 (2-tailed). P-values are unadjusted for multiple testing. The counts are for female cases and controls only but odds-ratios and results are from incorporating males and family history data - see online methods.

Supplementary Table 8**: Association results for PTVs or deleterious missense variants (CADD) and overall breast cancer.** All genes associated at P<0.001 in the meta-analysis are listed. Z-scores are from testing H0:β=ln(Odds Ratio)=0 (2-tailed)**.** P-values are unadjusted for multiple testing. The counts are for female cases and controls only but odds-ratios and results are from incorporating males and family history data - see online methods.

Supplementary Table 9: **Association results for PTVs or deleterious missense variants (Helix) and overall breast cancer. All** genes associated at P<0.001 in the meta-analysis are listed. Z-scores are from testing H0:β=ln(Odds Ratio)=0 (2-tailed)**.** P-values are unadjusted for multiple testing. The counts are for female cases and controls only but odds-ratios and results are from incorporating males and family history data - see online methods.

Supplementary Table 11: **Pathological characteristics for carriers of variants in genes with P<0.0001 in the meta-analysis of PTVs.** Samples with previously identified pathogenic mutations in BRCA1, BRCA2 and PALB2 were excluded.

Supplementary Table 12: **Pathological characteristics for carriers of variants with P<0.0001 for the meta-analysis of PTVs or predicted deleterious (CADD) rare missense variants**; excluding the known 5 genes – BRCA1, BRCA2, ATM, PALB2 and CHEK2.

Supplementary Table 13: **GSEA results for KEGG, REACTOME and BIOCARTA pathways**, **using gene rankings based on Z scores for the gene PTV burden meta-analysis.** All results with q-value<0.1 are listed.

Supplementary Table 18: **Association results for PTVs and overall breast cancer, with meta-analysis weights using the 5 known genes.** All genes associated at P<0.001 in the meta-analysis are listed. Z-scores are from testing H0:β=ln(Odds Ratio)=0 (2-tailed). All p-values are unadjusted for multiple testing.

Supplementary Methods

Variant calling in the Breast Cancer Association Consortium datasets

The same pipeline for variant calling was applied to both the BRIDGES and PERSPECTIVE data and followed the GATK (Genome Analysis Toolkit) best practices. Briefly, raw sequence data (FASTQ format) were pre-processed to produce BAM files. This involved alignment to the reference genome (hg38, downloaded from UCSC at http://hgdownload.cse.ucsc.edu/downloads.html#human) using the mem algorithm from bwa (v0.7.17; using the -M and -R parameters to mark shorter split hits as secondary and to update the BAM file header, respectively), the sorting of the reads using samtools sort (v1.10; with -m 1G -l 9 parameters) and their indexing using samtools index (v1.10). Identification and removal of duplicate read pairs from the same DNA fragments was performed using Picard's MarkDuplicates (v2.1.1; with the ASSUME_SORTED=true, REMOVE_DUPLICATES=true and the M= parameters). The base recalibration included the generation of a base quality score recalibration table with the GATK BaseRecalibrator software (v4.1.4.1; using the --known-sites parameter with the Mills and 1000G gold standard indels and the dbSNP v146 annotations files downloaded from GATK's resource bundle and -L parameter with the interval files provided with the library preparation kits), later applied to the read bases to adjust their quality scores and increase the accuracy of the variant calling algorithms with the GATK BQSR software (v4.1.4.1; using the previously produced recalibration table). An intermediate and informal QC was performed for a sanity check, including coverage and alignment mapping metrics using samtools flagstat software (v1.10) and Picard's (v2.22.2) CollectInsertSizeMetrics (M=0.5), CollectAlignmentSummaryMetrics , CollectGcBiasMetrics (with the CHART= and S= parameters), CollectQualityYieldMetrics, CollectSequencingArtifactMetrics, CollectMultipleMetrics and CollectHsMetrics (with BAIT_INTERVALS= and the TARGET_INTERVALS= parameters using the intervals files provided by the respective library preparation kits) tools. Variants were then called using GATK HaplotypeCaller (v4.1.4.1; with the -L parameter to specify the interval and the -ERC GVCF parameter) for the whole exome and the results were compiled in multiple databases using GATK GenomicsDBImport software (v4.1.4.1; with the -L parameter to specify the chromosome). Each database contained only the results of a single chromosome for the analysis due to file size constraints. The GATK GenotypeGVCFs (v4.1.4.1) tool was used for the joint genotyping step on each genomic database. The variants with an excess heterozygosity were filtered out using GATK VariantFiltration (v4.1.4.1; with the --filter-expression \"ExcessHet > 54.69\" and the --filter-name ExcessHet parameters) and the genotype information was removed prior to the recalibration using GATK MakeSitesOnlyVcf (v4.1.4.1). The GATK VariantRecalibrator (v4.1.4.1) software was used to produce tranches files on SNP and indel separately with the --trust-all-polymorphic, -tranche 100.0, -tranche, 99.95, -tranche 99.9, -tranche 99.8, -tranche 99.6, -tranche 99.5, -tranche 99.4, -tranche 99.3, -tranche 99.0, -tranche 98.0, -tranche 97.0, -tranche 90.0, --resource:hapmap,known=false,training=true,truth=true,prior=15.0 (for SNP only, with the Hapmap 3.3 file), --resource:omni,known=false,training=true,truth=false,prior=12.0 (for SNP only, with the 1000G omni v2.5 file), --resource:1000G,known=false,training=true,truth=false,prior=10.0 (for SNP only, with the 1000G phase1 high confidence file), --resource:mills,known=false,training=true,truth=true,prior=12.0 (for INDEL only, using the Mills and 1000G gold standard file), --resource:axiomPoly,known=false,training=true,truth=false,prior=10.0 (for INDEL only, using the Axiom Exome Plus all populations poly file), --resource:dbsnp,known=true,training=false,truth=false,prior=2.0 (for

SNP and INDEL, with the dbSNP v146 file), -an (QD, MQ, MQRankSum, ReadPosRankSum, FS, SOR and DP for SNP and QD, FS, SOR, DP, ReadPosRankSum and MQRankSum for INDEL), -mode (SNP or INDEL), --max-gaussians (6 for SNP and 4 for INDEL), --tranches-file and --rscript-file parameters. Unless specified otherwise, annotation files were downloaded from the GATK resource bundle. Finally, the tranches files were used to apply the recalibration using the GATK ApplyVQSR (v4.1.4.1; with the --truth-sensitivity-filter-level 99.9 and the --recal-file parameters).

Contribution of PTVs to the Familial Relative Risk

As described in the main methods we assume a prior distribution for effect sizes (log-odds ratio) in which a proportion, α, of genes are risk associated. For genes that are risk associated, the prior distribution for the log-relative risk is assumed to follow a negative exponential distribution. Thus, the log-relative risk β has a density of the form $f(\beta|\alpha,\eta)$:

$$\beta \sim \begin{cases} 0\ w.p.\ 1-\alpha \\ g(\beta|\eta)\ w.p.\ \alpha \end{cases}, \text{ where } g(\beta|\eta) \sim \eta \exp(-\eta\beta)$$

We derive an approximate likelihood for observed carrier counts separately for the BCAC dataset and UK biobank dataset, before combining them into one likelihood. This is maximised to estimate α, β and hence the posterior effect size distributions.

For the BCAC datasets, we consider a 2x2 contingency table of counts for each gene *j*:

	**Control**	**Case**	**Total**
**Non-carrier**	$N_{B0} - n_{B0j}$	$N_{B1} - n_{B1j}$	$N_{B0} + N_{B1} - n_{Bj}$
**Carrier**	n_{B0j}	n_{B1j}	$n_{B0j} + n_{B1j} = n_{Bj}$
	N_{B0}	N_{B1}	$N_{B0} + N_{B1}$

Given the relative risk e^{β_j} for gene j, and making the simplifying assumption that the frequency of pathogenic variants is low, the expected proportion of carriers that are cases is, to a good approximation, $P(Case\ |Carrier) = \frac{N_{B1}e^{\beta_j}}{N_{B0}+N_{B1}e^{\beta_j}}$.

Therefore, the number of case carriers, given the total number of carriers, can be modelled by a binomial distribution $n_{B1j} \sim Bin(n_{Bj}, \frac{N_{B1}e^{\beta_j}}{N_{B0}+N_{B1}e^{\beta_j}})$. Hence $P(n_{B1j}|\ n_{Bj}, \beta_j) = \binom{n_{Bj}}{n_{B1j}} \left(\frac{N_{B1}e^{\beta_j}}{N_{B0}+N_{B1}e^{\beta_j}}\right)^{n_{B1j}} \left(1 - \frac{N_{B1}e^{\beta_j}}{N_{B0}+N_{B1}e^{\beta_j}}\right)^{n_{B0j}}$. Defining $\gamma_B = log(\frac{N_{B1}}{N_{B0}})$, this simplifies to:

$$P(n_{B1j}|\ n_{Bj}, \beta_j) = \binom{n_{Bj}}{n_{B1j}} \frac{(e^{\gamma_B}+1)^{n_{B0j}+n_{B1j}} e^{\beta_j n_{B1j}}}{(e^{\beta_j+\gamma_B}+1)^{n_{B0j}+n_{B1j}}}$$

For the UK Biobank dataset, we stratify the carrier counts by sex and family history status:

	FEMALE					MALE				
	Control		Case			Control		Case		
	FH 0	FH 1	FH 0	FH 1		FH 0	FH 1	FH 0	FH 1	
**Non-carrier**	$N_{F0} - n_{F0j}$	$N_{F1} - n_{F1j}$	$N_{F2} - n_{F2j}$	$N_{F3} - n_{F3j}$	$N_F - n_F$	$N_{M0} - n_{M0j}$	$N_{M1} - n_{M1j}$	N_{M2}- $- n_{M2j}$	$N_{M3} - n_{M3j}$	$N_M - n_M$
**Carrier**	n_{F0j}	n_{F1j}	n_{F2j}	n_{F3j}	$n_{F0j} + n_{F1j} + n_{F2j} + n_{F3j} = n_F$	n_{M0j}	n_{M1j}	n_{M2j}	n_{M3j}	$n_{M0j} + n_{M1j} + n_{M2j} + n_{M3j} = n_M$
	N_{F0}	N_{F1}	N_{F2}	N_{F3}	$N_{F0} + N_{F1} + N_{F2} + N_{F3} = N_F$	N_{M0}	N_{M1}	N_{M2}	N_{M3}	$N_{M0} + N_{M1} + N_{M2} + N_{M3} = N_M$

Thus, for each sex (subscript F or M), the phenotype of an individual has four possibilities (control +/- family history, case +/- family history). The probabilities of these phenotypes for a carrier are given by:

$$P(phenotype = k|carrier) = \frac{N_k e^{\beta_k j}}{N_0 + N_1 e^{\beta_1 j} + N_2 e^{\beta_2 j} + N_3 e^{\beta_3 j}}$$

Where $e^{\beta_k j}$ is the relative risk of phenotype *k*, relative to phenotype 0. For a rare dominant disease allele, and assuming that positive family history is relatively rare, $e^{\beta_{1j}} \approx \frac{1}{2}(e^{\beta_j} + 1)$, $e^{\beta_{2j}} \approx e^{\beta_j}$, $e^{\beta_{3j}} \approx \frac{1}{2}(3e^{\beta_j} - 1)$. That is, $e^{\beta_{kj}} \approx \frac{1}{2}\left(ke^{\beta_j} + 2 - k\right)$ [2].
Therefore, for each sex, the number of carriers in each stratum can be modelled by a multinomial distribution with a probability mass function:

$$n_{oj}, n_{1j}, n_{2j}, n_{3j} \sim Multinom\left(n_j, \frac{N_0}{N_0+\frac{1}{2}N_1\mathrm{e}^{\beta_j}+\frac{1}{2}N_1+N_2e^{\beta_j}+\frac{3}{2}N_3\mathrm{e}^{\beta_j}-\frac{1}{2}N_3}, \frac{\frac{1}{2}N_1\mathrm{e}^{\beta_j}+\frac{1}{2}N_1}{N_0+\frac{1}{2}N_1\mathrm{e}^{\beta_j}+\frac{1}{2}N_1+N_2e^{\beta_j}+\frac{3}{2}N_3\mathrm{e}^{\beta_j}-\frac{1}{2}N_3}, \frac{N_2e^{\beta_j}}{N_0+\frac{1}{2}N_1\mathrm{e}^{\beta_j}+\frac{1}{2}N_1+N_2e^{\beta_j}+\frac{3}{2}N_3\mathrm{e}^{\beta_j}-\frac{1}{2}N_3}, \frac{\frac{3}{2}N_3\mathrm{e}^{\beta_j}-\frac{1}{2}N_3}{N_0+\frac{1}{2}N_1\mathrm{e}^{\beta_j}+\frac{1}{2}N_1+N_2e^{\beta_j}+\frac{3}{2}N_3\mathrm{e}^{\beta_j}-\frac{1}{2}N_3}\right)$$
.

So that:

$$P\left(n_{oj}, n_{1j}, n_{2j}, n_{3j} | n_j, \beta_j\right) = \frac{\prod_{k=0}^{3}\left(N_k\frac{1}{2}\left(ke^{\beta_j}+2-k\right)\right)^{n_{kj}}}{\left(\sum_{k=0}^{3} N_k\frac{1}{2}\left(ke^{\beta_j}+2-k\right)\right)^{n_j}}$$

Defining $\gamma_{Fk} = log(\frac{N_{Fk}}{N_{F0}}), \gamma_{Mk} = log(\frac{N_{Mk}}{N_{M0}})$, and multiplying the probabilities for males and females, this simplifies to:

$$P(n_{oj}, n_{1j}, n_{2j}, n_{3j}|n_j, \beta_j) = C\frac{\prod_{k=0}^{3}\left(\frac{1}{2}\left(ke^{\beta_j}+2-k\right)\right)^{n_{Fkj}}\prod_{k=0}^{3}\left(\frac{1}{2}\left(ke^{\beta_j}+2-k\right)\right)^{n_{Mkj}}}{\left(\sum_{k=0}^{3}e^{\gamma_{F_k}}\frac{1}{2}(ke^{\beta_j}+2-k)\right)^{n_{Fj}}\left(\sum_{k=0}^{3}e^{\gamma_{M_k}}\frac{1}{2}(ke^{\beta_j}+2-k)\right)^{n_{Mj}}}$$

Where $C = \binom{n_{Fj}}{n_{F0j} \quad n_{F1j} \quad n_{F2j} \quad n_{F3j}}\binom{n_{Mj}}{n_{M0j} \quad n_{M1j} \quad n_{M2j} \quad n_{M3j}}e^{\sum_{k=0}^{3}\gamma_{Fk}n_{Fkj}+\sum_{k=0}^{3}\gamma_{Mk}n_{Mkj}}$ is independent of the prior distribution.

The likelihoods for the BCAC dataset and the UK biobank dataset are then multiplied and integrated over the prior distribution to give the likelihood to be maximised:

$$L(\alpha,\eta) \propto \prod_{j=1}^{J}\int \frac{e^{\beta_j n_{B1j}}}{(e^{\beta_j+\gamma_B}+1)^{n_{B0j}+n_{B1j}}}\frac{\prod_{k=0}^{3}\left(\frac{1}{2}\left(ke^{\beta_j}+2-k\right)\right)^{n_{Fkj}}\prod_{k=0}^{3}\left(\frac{1}{2}\left(ke^{\beta_j}+2-k\right)\right)^{n_{Mkj}}}{\left(\sum_{k=0}^{3}e^{\gamma_{F_k}}\frac{1}{2}(ke^{\beta_j}+2-k)\right)^{n_{Fj}}\left(\sum_{k=0}^{3}e^{\gamma_{M_k}}\frac{1}{2}(ke^{\beta_j}+2-k)\right)^{n_{Mj}}}f(\beta_j|\alpha,\eta)d\beta_j$$

Where $f(\beta_j|\alpha,\eta)$ is the prior distribution on β_j.

Writing: $L_j(\beta_j) = (e^{\gamma_B}+1)^{n_{B0j}+n_{B1j}}\left(\sum_{k=0}^{3}e^{\gamma_{F_k}}\right)^{n_{Fj}}\left(\sum_{k=0}^{3}e^{\gamma_{M_k}}\right)^{n_{Mj}}\frac{e^{\beta_j n_{B1j}}}{\left(e^{\beta_j+\gamma_B}+1\right)^{n_{B0j}+n_{B1j}}}\frac{\prod_{k=0}^{3}\left(\frac{1}{2}\left(ke^{\beta_j}+2-k\right)\right)^{n_{Fkj}}\prod_{k=0}^{3}\left(\frac{1}{2}(ke^{\beta_j}+2-k)\right)^{n_{Mkj}}}{\left(\sum_{k=0}^{3}e^{\gamma_{F_k}}\frac{1}{2}\left(\left(ke^{\beta_j}+2-k\right)\right)\right)^{n_{Fj}}\left(\sum_{k=0}^{3}e^{\gamma_{M_k}}\frac{1}{2}(ke^{\beta_j}+2-k)\right)^{n_{Mj}}}$

$L(\alpha,\eta) \propto \prod_{j=1}^{J}\int L_j(\beta_j)f(\beta_j|\alpha,\eta)d\beta_j = \prod_{j=1}^{J}\left(1-\alpha+\alpha\int L_j(\beta_j)g(\beta_j|\eta)d\beta_j\right) = \prod_{j=1}^{J}(1-\alpha+\alpha L_{*j})$ say.

A major advantage of this approach, which conditions on the total carrier count, is that it avoids the problems of estimating the allele frequency for each gene simultaneously with the relative risk parameters so that only maximisation over $\alpha$and η is required. This is relatively straightforward.

The posterior probability a gene is associated, given the estimates of $\alpha$and η, is then:

$$P(\beta_j|Data) = \frac{\alpha \int L_j(\beta_j) g(\beta_j|\eta) d\beta_j}{1 - \alpha + \alpha \int L_j(\beta_j) g(\beta_j|\eta) d\beta_j} = \frac{\alpha L_{*j}}{1 - \alpha + \alpha L_{*j}}$$

The posterior mean β_j is given by:

$$\frac{\int \beta_j L_j(\beta_j) g(\beta_j|\eta) d\beta_j}{L_{*j}}$$

And the posterior mean relative risk e^{β_j} is given by:

$$\frac{\int e^{\beta_j} L_j(\beta_j) g(\beta_j|\eta) d\beta_j}{L_{*j}}$$

To calculate the contribution of PTVs to the FRR, we note that for genes with aggregate PTV frequency, p_j, associated with relative risk e^{β_j}, the FRR is:

$$\lambda_j = 1 + \frac{p_j(e^{\beta_j} - 1)^2}{(2p_j(e^{\beta_j} - 1) + 1)^2}$$

This requires an estimate of the allele frequency for each gene. The simplest approach is to use the carrier frequencies in controls, thus:

$$\hat{p}_{Aj} = \frac{(n_{F0j} + n_{F1j} + n_{B0j})}{2(N_{F0} + N_{F1} + N_{B0})}$$

Hence:

$$\lambda_{jA} = 1 + \frac{\alpha}{1 - \alpha + \alpha L_{*j}} \int L_j(\beta_j) g(\beta_j|\eta) \frac{p_{jA}(e^{\beta_j} - 1)^2}{(2p_{jA}(e^{\beta_j} - 1) + 1)^2} d\beta_j$$

A potentially better approach is to also utilise the case data, estimating the allele frequency based on the posterior distribution of the relative risk. Thus:

$$p_{Bj}(\beta_j) = \frac{n_{F0j} + n_{F1j} + n_{F2j} + n_{F3j} + n_{B0j} + n_{B1j}}{2(N_{F0} + N_{F1} + N_{B0} + e^{\beta_j}(N_{F2} + N_{F3} + N_{B1})}$$

We further adjusted this estimate to account for the structural number variant frequency in each gene. Let:

$P(CNV)_j =$ CNV frequency in gene j, as estimated in the UK biobank Whole Genome Sequencing data

$P(PTV)_j$ = PTV frequency in gene j, in the same group of individuals as used for estimating $P(CNV)_j$

Assuming the events are independent but not mutually exclusive:

$$P(PTV \cup CNV)_j = P(PTV)_j + P(CNV)_j - P(PTV \cap CNV)_j = P(PTV)_j + P(CNV)_j - P(PTV)_j P(CNV)_j$$

The percentage increase multiplier for the PTV frequency accounting for the CNV frequency in gene j is $\frac{P(PTV\ U\ CNV)_j}{P(PTV)_j}$
We scale $p_{Bj}(\beta_j)$ by this multiplier to give p'_{Bj}

Hence:

$$\lambda_{jB} = 1 + \frac{\alpha}{1 - \alpha + \alpha L_{*j}} \int L_j(\beta_j) g(\beta_j|\eta) \frac{p'_{jB}(\beta_j)(e^{p'_{jB}(\beta_j)} - 1)^2}{(2p'_{jB}(\beta_j)(e^{p'_{jB}(\beta_j)} - 1) + 1)^2} d\beta_j$$

In the main analyses, we used the second method. However, for *BRCA1, BRCA2* and *PALB2* replaced the allele frequency estimates with external estimates from the literature, namely the estimates used in the current default BOADICEA/Canrisk model[3]. This is to account for the fact that the allele frequency estimates in the dataset will be underestimated (due to the deliberate exclusion of known carriers in the case of the BCAC exome sequencing, and potential under-ascertainment of carriers in UK Biobank).

The total FRR over all genes, assuming an additive model, is then given by:

$$\hat{\lambda}_{TOT} = 1 + \sum_{j=1}^{J} (\lambda_j - 1)$$

Assuming that the PTVs combined multiplicatively with other genetic or familial factors, and an overall FRR of 2, the percentage contribution of each gene to the overall FRR is therefore: $100 \times \frac{log(\hat{\lambda}_j)}{log\ (2)}$ and the total contribution of PTVs in all genes is: $100 \times \frac{log(\hat{\lambda}_{TOT})}{log\ (2)}$.

References

1. Han, B. & Eskin, E. Random-Effects Model Aimed at Discovering Associations in Meta-Analysis of Genome-wide Association Studies. *The American Journal of Human Genetics* **88**, 586-598 (2011).
2. Risch, N. Linkage strategies for genetically complex traits. I. Multilocus models. *American journal of human genetics* **46**, 222-228 (1990).
3. Lee, A. *et al.* Enhancing the BOADICEA cancer risk prediction model to incorporate new data on RAD51C, RAD51D, BARD1, updates to tumour pathology and cancer incidences. (Cold Spring Harbor Laboratory, 2022).
