# Supplementary material for: Exome sequencing identifies breast cancer susceptibility genes and defines the contribution of coding variants to breast cancer risk
Source: Nat Genet. 2023 Aug 17;55(9):1435–9. doi: 10.1038/s41588-023-01466-z (PMC10484782; doi:10.1038/s41588-023-01466-z)
Supplement: Supplementary file 2 — Reporting Summary [file 41588_2023_1466_MOESM2_ESM.pdf]

nature portfolio

Corresponding author(s): Douglas Easton

Last updated by author(s): 03/03/2023

Reporting Summary

Nature Portfolio wishes to improve the reproducibility of the work that we publish. This form provides structure for consistency and transparency in reporting. For further information on Nature Portfolio policies, see our Editorial Policies and the Editorial Policy Checklist.

Please do not complete any field with "not applicable" or n/a. Refer to the help text for what text to use if an item is not relevant to your study.
For final submission: please carefully check your responses for accuracy; you will not be able to make changes later.

Statistics

For all statistical analyses, confirm that the following items are present in the figure legend, table legend, main text, or Methods section.

n/a	Confirmed	
☐	☒	The exact sample size (n) for each experimental group/condition, given as a discrete number and unit of measurement
☒	☐	A statement on whether measurements were taken from distinct samples or whether the same sample was measured repeatedly
☐	☒	The statistical test(s) used AND whether they are one- or two-sided<br>*Only common tests should be described solely by name; describe more complex techniques in the Methods section.*
☐	☒	A description of all covariates tested
☐	☒	A description of any assumptions or corrections, such as tests of normality and adjustment for multiple comparisons
☐	☒	A full description of the statistical parameters including central tendency (e.g. means) or other basic estimates (e.g. regression coefficient) AND variation (e.g. standard deviation) or associated estimates of uncertainty (e.g. confidence intervals)
☐	☒	For null hypothesis testing, the test statistic (e.g. F, t, r) with confidence intervals, effect sizes, degrees of freedom and P value noted<br>*Give P values as exact values whenever suitable.*
☐	☒	For Bayesian analysis, information on the choice of priors and Markov chain Monte Carlo settings
☒	☐	For hierarchical and complex designs, identification of the appropriate level for tests and full reporting of outcomes
☐	☒	Estimates of effect sizes (e.g. Cohen's d, Pearson's r), indicating how they were calculated

Our web collection on statistics for biologists contains articles on many of the points above.

Software and code

Policy information about availability of computer code

Data collection	Sequence data were demultiplexed, aggregated in BAM files and processed through a pipeline based on the Picard (v.2.1.1) suite of software tools and samtools (v.10). Reads were mapped onto human genome build 38 using BWA (v0.7.17). Variants were called using the Genome Analysis Toolkit (GATK; v4.1.4.1) HaplotypeCaller package to produce variant call set (VCF) files. Variants were further filtered using Variant Quality Score Recalibration (VQSR). Structural variants in UK Biobank were called using GraphTyper (2.7.1).
Data analysis	Quality Control filtering of vcf files was performed using vcftools v0.1.15, bcftools v1.9, picard v2.22.2 and plink v1.90b, as outlined in the methods. Variants were annotated using Ensembl Variant Effect Predictor v101 with assembly GRCh38. Structural variants were called using GraphTyper (). The code for each software is available at the website of each package. Data manipulation and analysis were performed using R-4.13 with packages clusterProfiler (4.2.2), data.table (1.14.2), dplyr (1.0.9), gtools (3.9.2.1), HGNChelper (0.8.9), msigdbr (7.5.1), tibble (3.1.7) and tidyr (1.2.0). Plots were created using R-4.13 using additional packages ggplot2 (3.3.6) and ggrepel (0.9.1). The code for each of the R packages can be found in their associated vignettes.

For manuscripts utilizing custom algorithms or software that are central to the research but not yet described in published literature, software must be made available to editors and reviewers. We strongly encourage code deposition in a community repository (e.g. GitHub). See the Nature Portfolio guidelines for submitting code & software for further information.

Data

Policy information about availability of data

All manuscripts must include a data availability statement. This statement should provide the following information, where applicable:

- Accession codes, unique identifiers, or web links for publicly available datasets
- A description of any restrictions on data availability
- For clinical datasets or third party data, please ensure that the statement adheres to our policy

Data for the BRIDGES and PERSPECTIVE studies are available on reasonable request via the BCAC Data Access Co-ordinating Committee (BCAC@medschl.cam.ac.uk). Requests for UK Biobank should be made to the UK Biobank Access Management Team.

Human research participants

Policy information about studies involving human research participants and Sex and Gender in Research.

Reporting on sex and gender	Overall numbers of female and male subjects have been presented. Both males and females were included but, as breast cancer was the primary disease of interest, the number of male cases was insufficient to consider separate analyses by sex. Individuals whose genetically determined sex differed from their self-reported sex or gender were excluded.
Population characteristics	Participant numbers are summarised in Supplementary Tables 1 and 2. Diagnoses: invasive or in-situ breast cancer and control subjects without breast cancer. Genetic ancestry: individuals from MYBRCA and SGBCC studies were of east Asian ancestry, individuals in the remaining BCAC studies and UK Biobank studies were of European ancestry. Age distribution: UK biobank subjects were aged 38 - 73 years at recruitment (mean 56.8) and 14-82 at diagnosis (mean 57.1) BCAC cases were 18 - 86 year at diagnosis (mean 47.4), BCAC controls were 18 - 84 (mean 54.4) at last observation. Cases selected for this project we preferentially selected for early age at onset and/or family history of breast cancer. Known carriers of pathogenic germline BRCA1, BRCA2 and PALB2 variants, at the time of sample selection, were excluded.
Recruitment	The recruitment of cases and controls for BCAC studies varied by contributing study. Some studies recruited through hospital clinics while others recruited through population-based cancer registries. Recruitment strategies are summarised in Supplementary Table 24. UK Biobank subjects were recruited from 22 sites across the UK, through NHS patient registers. Cases cases were identified through linkage to the national cancer registration systems and through self-report.
Ethics oversight	The organisations providing ethical approval for the contributing studies are summarised in Supplementary Table 20.

Note that full information on the approval of the study protocol must also be provided in the manuscript.

Field-specific reporting

Please select the one below that is the best fit for your research. If you are not sure, read the appropriate sections before making your selection.

☒ Life sciences ☐ Behavioural & social sciences ☐ Ecological, evolutionary & environmental sciences

Life sciences study design

All studies must disclose on these points even when the disclosure is negative.

Sample size	A total of 26,368 female cases, 217,673 female controls, 94 male cases and 191,820 male controls were included in the analysis after quality control exclusions. The aim was to utlise the largest available dataset in order to maximise the power to detect associations, so no sample size calculation was relevant.
Data exclusions	Data exclusions are detailed in the methods. Variants were excluded on the basis of low sequencing depth, allelic balance, missingness and deviation from Hardy-Weinberg equilibrium. Samples with missing calls for >15% were excluded. For closely related individuals, only one individual was retained. For UK Biobank, individuals with excess relatives in the dataset were excluded. For UK Biobank individuals of non-European ancestry were excluded while for BCAC, individual not of European or east Asian ancestry were excluded.
Replication	These association analyses, used all the available data - the results from the BCAC and UK Biobank were combined in a meta-analysis to maximise power. While the novel associated reached a stringent level of signficance, replication in additional datasets will be required.
Randomization	Not applicable, this is an observational study.
Blinding	This is not an experimental study and did not require blinding. Processing of the sequence data was conducted independently of phenotypic data.

Reporting for specific materials, systems and methods

We require information from authors about some types of materials, experimental systems and methods used in many studies. Here, indicate whether each material, system or method listed is relevant to your study. If you are not sure if a list item applies to your research, read the appropriate section before selecting a response.

Materials & experimental systems

n/a	Involved in the study
☒	☐ Antibodies
☒	☐ Eukaryotic cell lines
☒	☐ Palaeontology and archaeology
☒	☐ Animals and other organisms
☒	☐ Clinical data
☒	☐ Dual use research of concern

Methods

n/a	Involved in the study
☒	☐ ChIP-seq
☒	☐ Flow cytometry
☒	☐ MRI-based neuroimaging

This checklist template is licensed under a Creative Commons Attribution 4.0 International License, which permits use, sharing, adaptation, distribution and reproduction in any medium or format, as long as you give appropriate credit to the original author(s) and the source, provide a link to the Creative Commons license, and indicate if changes were made. The images or other third party material in this article are included in the article's Creative Commons license, unless indicated otherwise in a credit line to the material. If material is not included in the article's Creative Commons license and your intended use is not permitted by statutory regulation or exceeds the permitted use, you will need to obtain permission directly from the copyright holder. To view a copy of this license, visit http://creativecommons.org/licenses/by/4.0/
